# Genetic Variation in Autophagy-Related Genes Influences the Risk and Phenotype of Buruli Ulcer

**DOI:** 10.1371/journal.pntd.0004671

**Published:** 2016-04-29

**Authors:** Carlos Capela, Ange Dodji Dossou, Rita Silva-Gomes, Ghislain Emmanuel Sopoh, Michel Makoutode, João Filipe Menino, Alexandra Gabriel Fraga, Cristina Cunha, Agostinho Carvalho, Fernando Rodrigues, Jorge Pedrosa

**Affiliations:** 1 Life and Health Sciences Research Institute (ICVS), School of Health Sciences, University of Minho, Braga, Portugal; 2 ICVS/3B’s - PT Government Associate Laboratory, Braga/Guimarães, Portugal; 3 Buruli Ulcer Treatment Center of Allada, Allada, Benin; 4 Regional Institute for Public Health, Ouidah, Benin; Swiss Tropical and Public Health Institute, SWITZERLAND

## Abstract

**Introduction:**

Buruli ulcer (BU) is a severe necrotizing human skin disease caused by *Mycobacterium ulcerans*. Clinically, presentation is a sum of these diverse pathogenic hits subjected to critical immune-regulatory mechanisms. Among them, autophagy has been demonstrated as a cellular process of critical importance. Since microtubules and dynein are affected by mycolactone, the critical pathogenic exotoxin produced by *M*. *ulcerans*, cytoskeleton-related changes might potentially impair the autophagic process and impact the risk and progression of infection.

**Objective:**

Genetic variants in the autophagy-related genes *NOD2*, *PARK2* and *ATG16L1* has been associated with susceptibility to mycobacterial diseases. Here, we investigated their association with BU risk, its severe phenotypes and its progression to an ulcerative form.

**Methods:**

Genetic variants were genotyped using KASPar chemistry in 208 BU patients (70.2% with an ulcerative form and 28% in severe WHO category 3 phenotype) and 300 healthy endemic controls.

**Results:**

The rs1333955 SNP in *PARK2* was significantly associated with increased susceptibility to BU [odds ratio (OR), 1.43; P = 0.05]. In addition, both the rs9302752 and rs2066842 SNPs in *NOD2* gee significantly increased the predisposition of patients to develop category 3 (OR, 2.23; P = 0.02; and OR 12.7; P = 0.03, respectively, whereas the rs2241880 SNP in *ATG16L1* was found to significantly protect patients from presenting the ulcer phenotype (OR, 0.35; P = 0.02).

**Conclusion:**

Our findings indicate that specific genetic variants in autophagy-related genes influence susceptibility to the development of BU and its progression to severe phenotypes.

## Author Summary

Buruli ulcer (BU) is a neglected tropical disease caused by *Mycobacterium ulcerans*. Because the exact trigger is still under investigation, current treatment options rely mostly on the surgical excision of the affected site. There is therefore a pressing demand for improved risk prediction and tailored treatment as well as for new drug targets. By resorting to the largest case-control study reported to date, we show that genetic variation in the autophagy-related genes *NOD2*, *PARK2* and *ATG16L1* influence the risk and course of BU disease. Thus, our results provide crucial insights into the role of autophagy in the pathogenesis of BU.

## Introduction

Buruli ulcer (BU) is a severe necrotizing human skin disease caused by *Mycobacterium ulcerans*, representing the third most common mycobacteriosis worldwide [1]. At least 33 countries from Africa, South America and Western Pacific, with tropical, subtropical and temperate climates, have reported BU [1]. Moreover, in 2014, 2200 new cases were reported in 12 of those 33 countries [1]. BU initiates as a small, painless, raised skin papule, nodule, plaque or oedema. Later, destruction of the subcutaneous adipose tissue leads to collapse of the epidermis and formation of a characteristic ulcer with undermined edges [1]. Advanced lesions display massive tissue destruction induced by the action of the exotoxin mycolactone, a potent cytotoxic and immunosuppressive polyketide-derived macrolide released by *M*. *ulcerans* [2]. Clinically, presentation is a sum of these diverse pathogenic hits subjected to critical, mainly local, immune-regulatory mechanisms [3].

Among the many immunological mechanisms defining susceptibility to infection and its progression, autophagy has been demonstrated as a cellular process of critical importance to immunity to viral, bacterial and protozoan infections [4]. Autophagy is a regulated process contributing to the innate control of intracellular pathogens by triggering the autodigestion of cytoplasmic components and driving pathogen clearance. Autophagy is known to be dependent on microtubule cytoskeleton and dynein-driven transport, with dynein playing a role in the delivery of autophagosome contents to lysosomes during autophagosome-lysosome fusion [4]. Since microtubules and dynein are affected by mycolactone [5], cytoskeleton-related changes might potentially impair the autophagic process and impact the risk and progression of *M*. *ulcerans* infection.

The function of specific components of the autophagic machinery, namely nucleotide-binding oligomerization domain-containing 2 (NOD2), E3 ubiquitin-protein ligase parkin (PARK2) and autophagy-related protein 16–1 (ATG16L1), has been associated with resistance to several intracellular pathogens, including *M*. *tuberculosis* [4]. Based on reports linking variants in these genes with defective activation of autophagy as well as our own data proposing a central role for autophagy in the intracellular control of *M*. *ulcerans* infection through mycolactone-induced impairment of cytoskeleton-dependent cellular functions [5], we designed a case-control genetic association study involving 208 prospectively collected cases of BU to dissect the contribution of selected autophagy-related genes to the risk of disease and its distinct phenotypes.

## Materials and Methods

### Patients and study design

The study population comprised 508 individuals from Zé District (Atlantique Department, Benin), with 208 newly diagnosed BU patients recruited at the Centre de Deépistage et de Traitement de l’Ulceère de Buruli d’Allada after 2005, and 300 unrelated, age and gender-matched controls, with similar water contact habits and the same ethnic background (healthy endemic controls) [1] (Table 1). This area presents a high incidence of BU, low consanguinity and uniform ethnicity [6]. All the subjects enrolled were HIV-negative and BCG-vaccinated. Collection of patient-level data included age, gender, clinical form, number and location of lesions and World Health Organization (WHO) clinical classification—as a severity cataloguing. All the patients enrolled were diagnosed after 2005, were positive for at least two of the three WHO recommended diagnostic tests, and received appropriate treatment. The National Ethical Review Board of the Ministry of Health in Benin (IRB0006860) provided approval for this study (clearance Nu 018, 20/Oct/2011), and written informed consent was obtained from all adult participants. Parents or guardians provided informed consent on behalf of all child participants.

**Table 1:** General characteristics of Buruli ulcer (BU) patients and healthy controls.

Variable	BU (N = 208)	Controls (N = 300)	P value
**Age, median (range)**	14 (10–25)	17 (11–28)	0.247^a^
**Gender, no (%)**			
Male	119 (57)	154 (51)	0.206^b^
Female	89 (43)	146 (49)	
**Clinical form, no. (%)**			
Ulcer (± osteomyelitis)	146 (70.2)	-	-
Plaque	48 (23)	-	
Oedema	12 (6)	-	
Nodule	2 (1)	-	
**Site of lesion, no. (%)**			
Lower or upper limbs	181 (87)	-	-
Head or trunk	27 (13)	-	
**WHO category, no (%)**^c^			
1	38 (18)	-	-
2	112 (54)	-	
3	58 (28)	-	

^a^ P value is for Pearson’s χ2 test.

^b^ P value is for Mann-Whitney U test.

^c^ The WHO category of BU lesions was defined according to standard criteria as follows: category 1, maximum lesion diameter <5 cm; category 2, maximum lesion diameter 5–15 cm; and category 3, minimum lesion diameter >15 cm associated or not with osteomyelitis and/or multifocal lesions and/or at a critical site.

### Genotyping

Genomic DNA from whole blood samples from patients and donors was isolated using the NZY Blood gDNA Isolation kit (NZYTech) according to the manufacturer's instructions. SNPs were selected based on previous published evidence of association with susceptibility to other mycobacterial diseases (S1 Table), with a particular emphasis on genetic variants with well-described functional consequences. Specifically, genetic variants in the multi-step intracellular xenophagy recognition process of mycobacteria through the NOD2-ATG16L1 axis and the complementary parkin-mediated ubiquitination were selected, thereby reinforcing the probability to detect positive associations. Genotyping of *PARK2* (rs1333955, rs1040079, and rs1514343), *NOD2* (rs13339578, rs2066842, rs4785225, rs9302752, and rs5743278), and *ATG16L1* (rs2241880) SNPs was performed using the KASPar genotyping chemistry (LGC Genomics, UK) following the manufacturer’s instructions.

### Statistical analysis

The associations between SNPs and BU was performed using Pearson's χ2 test providing a value of odds ratio (OR) with a 95% confidence interval (CI) for different genetic models (co-dominant, dominant and recessive). A P value lower or equal to 0.05 was considered significant. The linkage disequilibrium (LD) and Hardy-Weinberg equilibrium (HWE) tests were assessed by using the Haploview 4.2 software. Genotype frequencies were used to phase the haplotype configurations by resorting to the same software.

## Results

A total of 208 newly diagnosed cases of BU and 300 unrelated controls were selected according to fulfillment criteria. Demographics and clinical features of cases and age- and gender-matched controls are summarized in Table 1. The median age of cases was 14 years [interquartile range (IQR): 10–25] and similar to that of controls [17 years (IQR: 11–28)]; P = 0.25. The gender distribution of cases and controls was also not significantly different [89 (43%) females in 208 cases; and 146 (49%) females in 300 controls; P = 0.21]. Clinical features were in concordance with general African characteristics of BU [1]. The dominant clinical form reported was the ulcer (70.2%, including 6 cases with osteomyelitis), the mainly affected site were the limbs (87%), and the WHO categories 1 to 3 were displayed in 18.3%, 53.8% and 27.9% of the cases, respectively. The minor allele frequencies and HWE values for all SNPs are shown in S1 Table.

**Fig 1:**
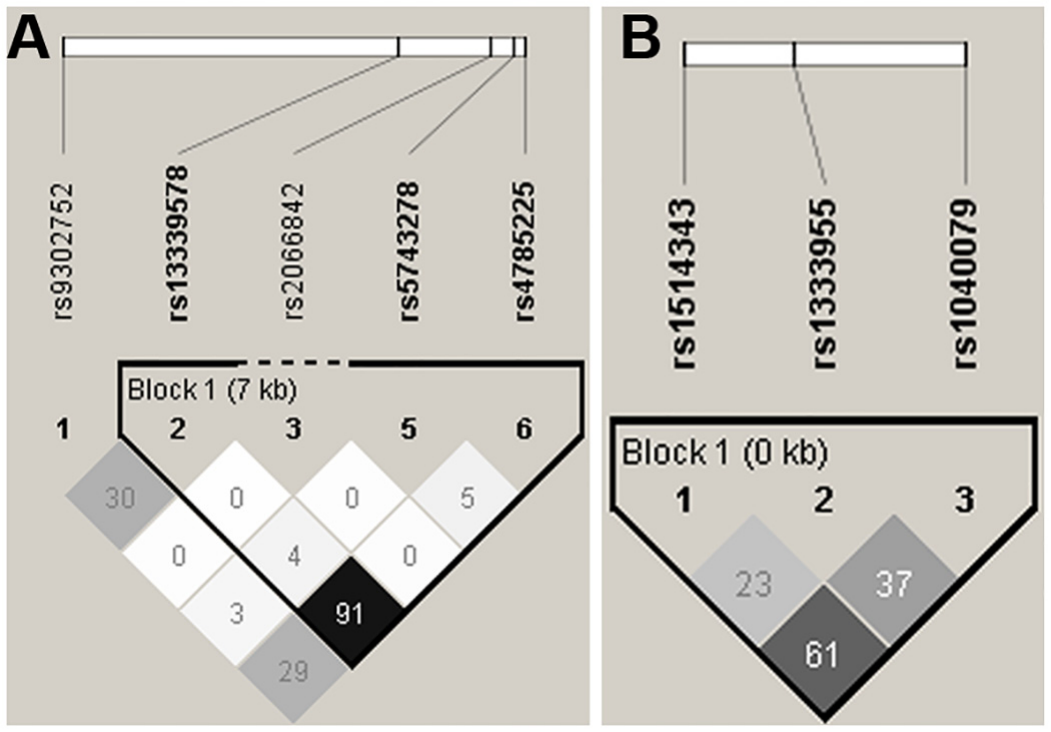
Haploview pairwise analysis of Linkage Disequilibrium (LD) between NOD2 gene SNPs (A) and PARK2 gene SNPs (B). The r2 colour scheme was used. Measures of r2 = 0 are represented in white (not significant); 0<r2<1 are represented in shades of grey; r2 = 1 are represented in black (significant). The numbers inside the box indicate the r2 in a percentage version. An r2 of 0 indicates complete linkage equilibrium, whereas an r2 of 1 indicates complete LD.

To assess the risk and progression of BU according to *NOD2*, *PARK2* and *ATG16L1* SNPs, we compared their genotype frequencies between BU patients and age- and gender-matched healthy controls. Whereas no significant variations in the distribution of genotypes among cases and controls were observed in the overall test of association, the rs1333955 SNP in the *PARK2* gene was significantly associated with increased susceptibility to BU upon modelling of a dominant mode of inheritance [OR, 1.43 (95% CI, 1.00–2.06); P = 0.05] (Table 2). Of interest, a similar though less significant association was also observed for patients carrying the rs1040079 SNP in the same gene [OR, 1.45 (95% CI, 0.96–2.18); P = 0.07]. Although the rs1333955 SNP was found to be in strong LD with rs1514343 and rs1040079 (Fig 1), none of the four haplotypes determined was significantly associated with the development of BU (S2 Table). No associations with the risk of BU were detected for SNPs in *NOD2* or *ATG16L1* (S3 Table). In addition, and although the rs13339578 SNP in the *NOD2* gene was in strong LD with both rs5743278 and rs4785225 SNPs (Fig 1), no associations were found for the haplotypes formed by this block (S4 Table).

**Table 2:** Genotype distributions and association test results of SNPs in the *PARK2* gene among BU patients and age- and gender-matched healthy controls.

Gene	SNP rs# Number	Alleles: status ^a^	Genotype, n (%) ^b^			P value ^c^		
			A/A	A/a	a/a	Overall	Recessive model	Dominant model
*PARK2*	rs1333955	C>T						
		BU	107 (52.2)	83 (40.5)	15 (7.3)	0.113	0.24	0.05
		Controls	177 (61.0)	99 (34.2)	14 (4.8)			
	rs1040079	G>A						
		BU	90 (30.4)	148 (50.0)	58 (19.6)	0.161	0.22	0.07
		Controls	47 (23.2)	107 (52.7)	49 (24.1)			
	rs1514343	G>A						
		BU	70 (33.7)	109 (52.4)	29 (13.9)	0.314	0.13	0.78
		Controls	97 (32.4)	145 (48.5)	57 (19.1)			

^a^ The first nucleotide represents the major allele.

^b^ Genotypes were defined according to the major (A) and minor (a) alleles at each SNP.

^c^ Association tests for the overall association (A/A vs. A/a vs. a/a), and the recessive (A/A + A/a vs. a/a) and dominant (A/A vs. A/a + a/a) genetic models were carried out using Fisher’s exact t test.

Since the clinical presentation of BU varies dramatically and epidemiological data has pointed out that host genetic factors may be involved in these phenotypes [1], we further evaluated the genetic susceptibility to the severe WHO category 3 or the ulcerative form of BU. We found that both the rs9302752 and rs2066842 (P268S) SNPs in the *NOD2* gene significantly increased the predisposition of patients to develop category 3 lesions following a dominant genetic model [OR, 2.23 (95% CI, 1.14–4.37); P = 0.02; and OR, 12.7 (95% CI, 0.60–269); P = 0.03), respectively] (Table 3). None of the other SNPs in *NOD2*, *PARK2* or *ATG16L1* revealed association with WHO category 3 (S5 Table). In what regards susceptibility to the ulcerative form of BU disease, the rs2241880 (T300A) SNP in the *ATG16L1* gene was found to significantly protect patients from presenting the ulcer phenotype when a recessive genetic model was applied [OR, 0.35 (95% CI, 0.13–0.90); P = 0.02] (Table 3). None of the other SNPs in *PARK2* or *NOD2* genes revealed associations with the degree of ulceration (S5 Table).

**Table 3:** Genotype distributions and association test results of SNPs in the *NOD2* and *ATG16L1* genes with the severe WHO category 3 or the ulcerative form of BU disease.

Gene	SNP rs# number	Alleles^a^: status	Genotype, n (%)^b^			P value^c^		
			A/A	A/a	a/a	Overall	Recessive model	Dominant model
*NOD2*	rs9302752	C>T						
		Cat. 1 or 2	63 (43.8)	60 (41.7)	21 (15.6)	0.05	0.64	0.02
		Cat. 3	15 (25.9)	33 (56.9)	10 (17.2)			
		Non-ulcerative	24 (38.7)	28 (45.2)	10 (16.1)	1.00	0.86	0.94
		Ulcerative	57 (39.3)	66 (45.5)	22 (15.2)			
	rs13339578	A>G						
		Cat. 1 or 2	42 (29.2)	68 (47.2)	34 (23.6)	0.54	0.27	0.82
		Cat. 3	16 (27.6)	24 (41.4)	18 (31.0)			
		Non-ulcerative	17 (27.4)	27 (43.6)	18 (29.0)	0.76	0.46	0.67
		Ulcerative	44 (30.4)	66 (45.5)	35 (24.1)			
	rs2066842	C>T						
		Cat. 1 or 2	143 (100)	0 (0.0)	0 (0.0)	0.08	1.00	0.03
		Cat. 3	56 (96.6)	2 (3.4)	0 (0.0)			
		Non-ulcerative	62 (100)	0 (0.0)	0 (0.0)	1.00	1.00	0.35
		Ulcerative	142 (98.6)	2 (1.4)	0 (0.0)			
	rs5743278	C>G						
		Cat. 1 or 2	131 (90.3)	14 (9.7)	0 (0.0)	0.35	0.11	0.82
		Cat. 3	53 (91.4)	4 (9.1)	1 (1.7)			
		Non-ulcerative	54 (87.1)	7 (11.3)	1 (1.6)	0.21	0.12	0.29
		Ulcerative	134 (91.8)	12 (8.2)	0 (0.0)			
	rs47885225	G>C						
		Cat. 1 or 2	41 (28.5)	68 (47.2)	35 (24.3)	0.60	0.33	0.90
		Cat. 3	16 (27.6)	24 (41.4)	18 (31.0)			
		Non-ulcerative	16 (25.8)	28 (45.2)	18 (29.0)	0.73	0.53	0.51
		Ulcerative	44 (30.3)	65 (44.9)	36 (24.8)			
*ATG16L1*	rs2241880	T>C						
		Cat. 1 or 2	76 (52.8)	55 (38.2)	13 (9.0)	0.36	0.74	0.17
		Cat. 3	24 (42.1)	27 (47.4)	6 (10.5)			
		Non-ulcerative	27 (43.6)	25 (40.3)	10 (16.1)	0.07	0.02	0.22
		Ulcerative	76 (52.8)	59 (41.0)	9 (6.2)			

^a^ The first nucleotide represents the major allele.

^b^ Genotypes were defined according to the major (A) and minor (a) alleles at each SNP.

^c^ Association tests for the overall association (A/A vs. A/a vs. a/a), and the recessive (A/A + A/a vs. a/a) and dominant (A/A vs. A/a + a/a) genetic models were carried out using Fisher’s exact t test.

## Discussion

We compared the prevalence of SNPs in autophagy-related genes in confirmed cases of BU and in randomly selected community controls equally exposed to similar risk factors such as relationship and same behaviors (recreational or not) related to stagnant waters around villages.

We found that the rs1333955 SNP in the *PARK2* gene was significantly associated with development of BU. The PARK2 protein—known as parkin—is associated with the process of protein ubiquitination, acting as an E3 ligase and targeting proteins for proteasomal degradation [7]. The ubiquitin-mediated pathway is a complementary system for autophagy activation and that contributes to pathogen elimination, including *M*. *tuberculosis*, by surrounding bacteria with conjugated ubiquitin chains. Our findings support a role for the *PARK2*/*PACRG* gene cluster in susceptibility to *M*. *ulcerans* infection, suggesting that mechanisms linked to ubiquitination and proteasome-mediated protein degradation might unveil a common pathway in the intracellular fate of this pathogen. The fact that the same SNP has been associated with a higher risk of leprosy [8] points to a pertinent role for this gene in both infections. In addition, PACRG has been suggested to preferentially bind to hydrophobic molecules, such as lipids [9]. Mycolactone, a lipid mycotoxin, was recently shown to inhibit translocation of newly translated proteins into the endoplasmic reticulum [10], culminating in their degradation by the proteasome. Accordingly, we have recently reported that mycolactone induces an increased amount of ubiquitinated proteins in the cell by affecting cytoskeleton constituents and cytoskeleton-dependent intracellular trafficking [5]. Ultimately, this points to likely critical consequences of the rs1333955 SNP on the proteasomal degradation induced by mycolactone and might explain, at least in part, its association with risk of BU.

Because autophagy is a pivotal immunological mechanism mediating protection to infection by intracellular pathogens [4], mycolactone-induced impairment of autophagy might have implications for the progression of BU disease. Previous studies have revealed that the NOD2-ATG16L1 axis is important for maintaining intracellular immune homeostasis [11]. The rs9302752 and rs2066842 SNPs in the *NOD2* gene were found to be significantly associated with a severe phenotype of BU disease, reflected by the WHO Category 3, suggesting a crucial role of genetic variability of the *NOD2* locus in defining severity of BU disease. The rs9302752 SNP is located in the upstream region of the gene, and therefore it might deregulate promoter activity and influence gene expression and susceptibility to infection. Indeed, silencing *NOD2* expression in human macrophages was reported to result in a local spread of *M*. *tuberculosis*, with an impairment in NOD2-mediated production of cytokines [12]. In addition, the rs2066842 SNP underlies the P268S amino acid substitution, and has been found to affect host recognition of bacterial muramyl dipeptide. As such, we hypothesize that a failure in the innate immune recognition of *M*. *ulcerans* via NOD2 might divert the proper activation of immunological autophagy, therefore permitting progression of infection and development of more severe phenotypes.

The non-ulcerative and ulcerative forms of BU can be observed as stable clinical phenotypes, and not all patients progress to the latter [1]. We found the rs2241880 SNP in the *ATG16L1* gene to be associated with protection of BU patients from an ulcerative clinical form. ATG16L1 is a master regulator of the core autophagy machinery that was initially identified as a pivotal risk factor for Crohn’s disease [13]. The rs2241880 variant is located in the coding region of *ATG16L1* and leads to the Thr300Ala (T300A) amino acid substitution, which has recently been found to enhance its self-degradation by caspase 3, thereby impairing autophagy activation [14]. Of interest, the T300A variant also decreased selective autophagy, resulting in increased interleukin (IL)-1β signaling and decreased antibacterial defense [14]. Increased levels of IL-1β are also associated with a more exuberant local inflammation. Indeed, non-ulcerative forms of BU, such as edema and plaque, are considered more inflammatory than ulcerative lesions [15].

Our study has some limitations. In particular, the study was conducted in a single population, and therefore it requires confirmation in larger groups and independent cohorts, as well as the assessment of the functional consequences of the associated variants and their influence to the immune response dynamics. It is however important to note that our case-control study has a robust sample size and the critical advantage that controls were carefully matched to cases regarding environmental exposure to mycobacteria.

Our findings indicate that specific genetic variants in autophagy-related genes influence susceptibility to the development of BU and its progression to severe phenotypes, highlighting the multiple additive effects of single genetic factors and their complex interactions towards the overall weight of the human immune response to *M*. *ulcerans*. Ultimately, this study reinforces the applicability of host genomics as an important factor to be considered in the stratification of infection risk in endemic regions and, more importantly, for the definition of patient groups more likely to advance to more severe and debilitating phenotypes of BU disease.

## Supporting Information

S1 Table Description of *NOD2*, *PARKIN2* and *ATG16L1* SNPs evaluated in BU patients and healthy controls. (DOCX)

S2 Table Haplotype frequencies and association test results in the PARK2 gene among BU patients and age- and gender-matched healthy controls. (DOCX)

S3 Table Genotype distributions and association test results of SNPs in the *NOD2* and *ATG16L1* genes among BU patients and age- and gender-matched healthy controls. (DOCX)

S4 Table Haplotype frequencies and association test results in the *NOD2* gene among BU patients and age- and gender-matched healthy controls. (DOCX)

S5 Table Genotype distributions and association test results of SNPs in the *PARK2* gene with the severe WHO category 3 or the ulcerative form of BU disease. (DOCX)
