# Supplementary material for: Genetic Variation in Autophagy-Related Genes Influences the Risk and Phenotype of Buruli Ulcer
Source: PLoS Negl Trop Dis. 2016 Apr 29;10(4):e0004671. doi: 10.1371/journal.pntd.0004671 (PMC4851401; doi:10.1371/journal.pntd.0004671)
Supplement: S1 Table — (DOCX) [file pntd.0004671.s001.docx]

**Table S1 – Description of *NOD2*, *PARKIN2* and *ATG16L1* SNPs evaluated in BU patients and healthy controls.**

| **Gene** | **SNP rs# number** | **Chromosome position** | **Alleles** | **Gene location** | **aa change** | **HapMap MAF** | **MAF in our study** | **HWE** |
| --- | --- | --- | --- | --- | --- | --- | --- | --- |
| *NOD2* | rs9302752 | 50719103 | C>T | Near gene 5’ | - | 0.412 | 0.378 | 0.55 |
|  | rs13339578 | 50739105 | A>G | Intron | - | 0.441 | 0.483 | 0.83 |
|  | rs2066842 | 50744624 | C>T | Missense | P268S | 0.009 | 0.008 | 1.00 |
|  | rs5743278 | 50745996 | C>G | Missense | A725G | 0.083 | 0.053 | 1.00 |
|  | rs4785225 | 50746546 | G>C | Intron | - | 0.455 | 0.484 | 0.96 |
| *PARK2* | rs1514343 | 163213083 | G>A | Intron | - | - | 0.420 | 0.60 |
|  | rs1333955 | 163213454 | C>T | Intron | - | 0.228 | 0.242 | 1.00 |
|  | rs1040079 | 163214027 | G>A | Intron | - | - | 0.470 | 0.64 |
| *ATG16L1* | rs2241880 | 233274722 | T>C | Missense | T300A | 0.270 | 0.297 | 0.10 |

SNP – single nucleotide polymorphism; aa – amino acid; MAF – minor allele frequency; HWE – Hardy-Weinberg equilibrium.

Chromosome positions are from NCBI database, assembly GRCh37.p13. HapMap MAFs were identified in the HapMap-YRI population.
