# Supplementary material for: Genetic Variation in Autophagy-Related Genes Influences the Risk and Phenotype of Buruli Ulcer
Source: PLoS Negl Trop Dis. 2016 Apr 29;10(4):e0004671. doi: 10.1371/journal.pntd.0004671 (PMC4851401; doi:10.1371/journal.pntd.0004671)
Supplement: S2 Table — (DOCX) [file pntd.0004671.s002.docx]

**Table S2.** Haplotype frequencies and association test results in the *PARK2* gene among BU patients and age- and gender-matched healthy controls.

| **Haplotype**^a^ | **Alleles**^b^**:status** | **Frequency (%)** | **P value**^c^ |
| --- | --- | --- | --- |
|  |  |  |  |
| H1 | A-C-G  BU  Controls | 0.396  0.430 | 0.29 |
| H2 | G-T-A |  |  |
|  | BU | 0.273 | 0.11 |
|  | Controls | 0.229 |  |
| H3 | G-C-A |  |  |
|  | BU | 0.224 | 0.74 |
|  | Controls | 0.216 |  |
| H4 | G-C-G |  |  |
|  | BU | 0.099 | 0.22 |
|  | Controls | 0.123 |  |

^a^ Haplotypes with frequencies ˃0.05 in either BU patients or controls are shown.

^b^ Pairwise linkage disequilibrium (LD) blocks were defined using the confidence intervals method. Genotype frequencies of the selected SNPs were used to phase haplotype configuration.

^c^ P values were calculated based on approximate chi-square distribution.
