# Supplementary material for: Genetic Variation in Autophagy-Related Genes Influences the Risk and Phenotype of Buruli Ulcer
Source: PLoS Negl Trop Dis. 2016 Apr 29;10(4):e0004671. doi: 10.1371/journal.pntd.0004671 (PMC4851401; doi:10.1371/journal.pntd.0004671)
Supplement: S3 Table — (DOCX) [file pntd.0004671.s003.docx]

**Table S3.** Genotype distributions and association test results of SNPs in the *NOD2* and *ATG16L1* genes among BU patients and age- and gender-matched healthy controls.

| **Gene** | **SNP rs# number** | **Alleles: status ^a^** | **Genotype, n (%) ^b^** | | |  | **P value ^c^** | | |
| --- | --- | --- | --- | --- | --- | --- | --- | --- | --- |
|  |  |  | **A/A** | **A/a** | **a/a** |  | **Overall** | **Recessive model** | **Dominant model** |
| *NOD2* | rs13339578 | A˃G  BU  Controls | 61 (29.5)  76 (25.3) | 93 (44.9)  157 (52.4) | 53 (25.6)  67 (22.3) |  | 0.263 | 0.39 | 0.30 |
|  | rs2066842 | C˃T |  |  |  |  |  |  |  |
|  |  | BU | 204 (99.0) | 2 (1.0) | 0 (0.0) |  | 0.476 | 1.00 | 0.31 |
|  |  | Controls | 273 (97.8) | 6 (2.0) | 0 (0.0) |  |  |  |  |
|  | rs4785225 | G˃C |  |  |  |  |  |  |  |
|  |  | BU | 60 (29.0) | 93 (44.9) | 54 (26.1) |  | 0.128 | 0.21 | 0.28 |
|  |  | Controls | 74 (24.7) | 162 (54.0) | 64 (21.3) |  |  |  |  |
|  | rs9302752 | C˃T |  |  |  |  |  |  |  |
|  |  | BU | 81 (39.1) | 94 (45.4) | 32 (15.5) |  | 0.984 | 0.82 | 0.94 |
|  |  | Controls | 118 (39.5) | 137 (45.8) | 44 (14.7) |  |  |  |  |
|  | rs5743278 | C˃G |  |  |  |  |  |  |  |
|  |  | BU | 188 (90.4) | 19 (9.1) | 1 (0.5) |  | 0.403 | 0.23 | 0.62 |
|  |  | Controls | 267 (89.0) | 33 (11.0) | 0 (0.0) |  |  |  |  |
| *ATG16L1* | rs2241800 | T˃C |  |  |  |  |  |  |  |
|  |  | BU | 103 (50.0) | 84 (40.8) | 19 (9.2) |  | 0.144 | 0.13 | 0.41 |
|  |  | Controls | 138 (46.3) | 143 (48.0) | 17 (5.7) |  |  |  |  |

^a^ The first nucleotide represents the major allele.

^b^ Genotypes were defined according to the major (A) and minor (a) alleles at each SNP.

^c^ Association tests for the overall association (A/A vs. A/a vs. a/a), and the recessive (A/A + A/a vs. a/a) and dominant (A/A vs. A/a + a/a) genetic models were carried out using Fisher’s exact t test.
