# Supplementary material for: Genetic Variation in Autophagy-Related Genes Influences the Risk and Phenotype of Buruli Ulcer
Source: PLoS Negl Trop Dis. 2016 Apr 29;10(4):e0004671. doi: 10.1371/journal.pntd.0004671 (PMC4851401; doi:10.1371/journal.pntd.0004671)
Supplement: S5 Table — (DOCX) [file pntd.0004671.s005.docx]

**Table S5.** Genotype distributions and association test results of SNPs in the *PARK2* gene with the severe WHO category 3 or the ulcerative form of BU disease.

| **Gene** | **SNP rs# number** | **Alleles^a^: status** | **Genotype, n (%)^b^** | | |  |  | **P value^c^** |  |
| --- | --- | --- | --- | --- | --- | --- | --- | --- | --- |
|  |  |  | **A/A** | **A/a** | **a/a** |  | **Overall** | **Recessive model** | **Dominant model** |
| *PARK2* | rs1514343 | C˃T |  |  |  |  |  |  |  |
|  |  | Cat. 1 or 2 | 47 (32.4) | 78 (53.8) | 20 (13.8) |  | 0.56 | 1.00 | 0.33 |
|  |  | Cat. 3 | 23 (39.7) | 27 (46.5) | 8 (13.8) |  |  |  |  |
|  |  | Non-ulcerative | 25 (40.3) | 30 (48.4) | 7 (12.3) |  | 0.40 | 0.47 | 0.18 |
|  |  | Ulcerative | 45 (30.8) | 79 (54.1) | 22 (15.1) |  |  |  |  |
|  | rs133955 | G˃A |  |  |  |  |  |  |  |
|  |  | Cat. 1 or 2 | 78 (54.9) | 56 (39.4) | 8 (5.6) |  | 0.82 | 0.12 | 0.13 |
|  |  | Cat. 3 | 107 (52.2) | 83 (40.5) | 15 (7.3) |  |  |  |  |
|  |  | Non-ulcerative | 29 (47.6) | 26 (42.6) | 6 (9.8) |  | 0.54 | 0.37 | 0.39 |
|  |  | Ulcerative | 78 (54.2) | 57 (39.6) | 9 (6.2) |  |  |  |  |
|  | rs1040079 | G˃A |  |  |  |  |  |  |  |
|  |  | Cat. 1 or 2 | 33 (23.6) | 74 (52.8) | 33 (23.6) |  | 0.82 | 0.55 | 0.86 |
|  |  | Cat. 3 | 13 (22.4) | 29 (50.0) | 16 (27.6) |  |  |  |  |
|  |  | Non-ulcerative | 15 (24.2) | 29 (46.8) | 18 (29.0) |  | 0.50 | 0.32 | 0.80 |
|  |  | Ulcerative | 32 (22.5) | 78 (55.0) | 32 (22.5) |  |  |  |  |

^a^ The first nucleotide represents the major allele.

^b^ Genotypes were defined according to the major (A) and minor (a) alleles at each SNP.

^c^ Association tests for the overall association (A/A vs. A/a vs. a/a), and the recessive (A/A + A/a vs. a/a) and dominant (A/A vs. A/a + a/a) genetic models were carried out using Fisher’s exact t test.
